# Assessment of triglyceride and cholesterol in overweight people based on multiple linear regression and artificial intelligence model

**DOI:** 10.1186/s12944-017-0434-5

**Published:** 2017-02-20

**Authors:** Jing Ma, Jiong Yu, Guangshu Hao, Dan Wang, Yanni Sun, Jianxin Lu, Hongcui Cao, Feiyan Lin

**Affiliations:** 1 Department of Laboratory Medicine, First Affiliated Hospital, College of Medicine, Zhejiang University, Key Laboratory of Clinical In Vitro Diagnostic Techniques of Zhejiang Province, Hangzhou, China; 2 The State Key Laboratory for Diagnosis and Treatment of Infectious Diseases, First Affiliated Hospital, College of Medicine, Zhejiang University, Collaborative Innovation Center for Diagnosis and Treatment of Infectious Diseases, 79 Qingchun Rd., Hangzhou City, 310003 China; 3 Key Laboratory for Laboratory Medicine of Ministry of Education, Wenzhou Medical University, Wenzhou, Zhejiang China; 4 Central laboratory, The First Affiliated Hospital of Wenzhou Medical University, Nanbaixiang street, Ouhai District, Wenzhou, 325000 China

**Keywords:** Triglyceride, Cholesterol, Overweight, Regression, Back propagation artificial neural network

## Abstract

**Background:**

The prevalence of high hyperlipemia is increasing around the world. Our aims are to analyze the relationship of triglyceride (TG) and cholesterol (TC) with indexes of liver function and kidney function, and to develop a prediction model of TG, TC in overweight people.

**Methods:**

A total of 302 adult healthy subjects and 273 overweight subjects were enrolled in this study. The levels of fasting indexes of TG (fs-TG), TC (fs-TC), blood glucose, liver function, and kidney function were measured and analyzed by correlation analysis and multiple linear regression (MRL). The back propagation artificial neural network (BP-ANN) was applied to develop prediction models of fs-TG and fs-TC.

**Results:**

The results showed there was significant difference in biochemical indexes between healthy people and overweight people. The correlation analysis showed fs-TG was related to weight, height, blood glucose, and indexes of liver and kidney function; while fs-TC was correlated with age, indexes of liver function (*P* < 0.01). The MRL analysis indicated regression equations of fs-TG and fs-TC both had statistic significant (*P* < 0.01) when included independent indexes. The BP-ANN model of fs-TG reached training goal at 59 epoch, while fs-TC model achieved high prediction accuracy after training 1000 epoch.

**Conclusions:**

In conclusions, there was high relationship of fs-TG and fs-TC with weight, height, age, blood glucose, indexes of liver function and kidney function. Based on related variables, the indexes of fs-TG and fs-TC can be predicted by BP-ANN models in overweight people.

**Electronic supplementary material:**

The online version of this article (doi:10.1186/s12944-017-0434-5) contains supplementary material, which is available to authorized users.

## Background

A survey of World Health Organization (WHO) completed in 2014 revealed there 39% of adults aged 18 years and over were overweight in the world. In China, the overweight people have increased from 1.8 to 11.9% in the period from 1985 to 2005 [1]. A cross-sectional survey of adolescents (ages 18–25) in American showed the rates of overweight and obesity (BMI >/= 25) were 21.3% and 10.8% [2]. In Europe in 2010 the highest prevalence of obesity among adults aged >15 years for men (30%) in Greece, and for women in Greece and the United Kingdom (26%) [3].

The high dyslipidaemia is an elevation of lipids in the blood, that is, elevation of triglyceride (TG), cholesterol (TC) and/or fat phospholipids. The elevated serum TG and/or TC are associated with a series of symptomatic vascular disease such as peripheral arterial and coronary artery disease, atherogenic lipoprotein phenotype, hypertension, insulin resistance, and glucose intolerance, and [4–7]. High level of TG (>11.3 mmol/L]) can cause acute pancreatitis, the TG level serve as an important indicator to determine the prognosis of patients with acute biliary pancreatitis [8, 9]. TG to high-density lipoprotein cholesterol ratio > or = 3.0 (in mg/dl) is a marker of insulin resistance in overweight [10].

There are no direct symptoms in the vast majority of cases with high TG and TC, the blood test is the only way to diagnose whether the TG and TC is too high. In clinical practice, TG and TC, blood glucose, liver function, and kidney function are determined independently. Commonly, few people will monitor them simultaneously. If we can find the relevance between the TG, TC with blood glucose, liver function, and kidney function. It will be convenient for us to assess the level of TG and TC when we had a test of blood glucose, liver function, and kidney function.

Being overweight is at high risk for high dyslipidaemia. Therefore, monitoring and prediction of TG and TC level is necessary in overweight people for early discovery and timely treatment. In this study, 346 subjects (254 male and 92 female) overweight people were analyzed by correlation analysis and multiple linear regression (MRL). In order to calculate the level of TG and TC, a back propagation artificial neural network (BP-ANN) model of TG and TC was developed in overweight people.

BP-ANN is a kind of artificial neural network, which is composed of input layer, hidden layer, and output layer [11]. Input layer represents the raw data, hidden layer accepts data from the input layer and modifies them using some algorithm. After processed in hidden layer, the new information is sent to the output layer. It is generally presented as systems of interconnected “neurons” which exchange messages between each other. The connections between input, hidden, and output layer have numeric weights that can be tuned and making neural nets adaptive to inputs and capable of learning [12]. BP-ANN has been widely used in medical field, such as analyzing the risk factors of diabetes mellitus, prediction of hemoglobin levels, Alzheimer’s disease screening [13–15].

## Methods

### Subjects

This study was approved by the First Affiliated Hospital of Wenzhou Medical University and conducted in accordance with the Declaration of Helsinki. The participants included in this study were divided into healthy people and overweight people according to the BMI (25.0–30 kg/m2) recommended by World Health Organization. The subject’s height was measured by using a stadiometer and weight using an electronic weighing scale with the participant wearing foam slippers and a shirt and pants. The subjects were excluded from this study if they had any history of diseases, such as hematopathy, angiocardiopathy, hepatopathy, nephrosis and pulmonary diseases.

### Blood biochemistry examination

The participants were randomly assigned to a clinical laboratory at physical examination center. A total of 3 mL fasting venous blood were collected into separation gel tubes and separated in a desk centrifuge at 5000 r/min for 10 min. After that, the plasma was analyzed at Hitachi 705/717 biochemical measurement. The fasting (fs) biochemical indexes of liver function, kidney function, blood glucose (fs-GLU), triglyceride (fs-TG), and total cholesterol (fs-TC) were determined. The indexes of liver function included: alanine transaminase (fs-ALT), aspartate aminotransferase (fs-ALT), gamma-glutamyl transpeptidase(fs-GGT), total protein (fs-TP), albumin (fs-ALB), alkaline phosphatase (fs-AKP), total bilirubin (fs-TBIL), and direct bilirubin (fs-DBIL). The indexes of kidney function were creatinine (fs-Cr), urea nitrogen (fs-BUN), and Uric Acid (fs-UA).

### Statistical analysis

According to the value of BMI, all subjects were divided into two groups, healthy people and overweight people. The distribution characteristic of the measuring data was analyzed by Shapiro-Wilk test and homogeneity test of variances. The difference of biochemical indexes between healthy people and overweight people was analyzed according to the normal distribution test. The relationships of fs-TG and fs-TC level with liver, kidney and fs-GLU were analyzed by bivariate correlation. MRL analysis was used to identify factors related to the fs-TG and fs-TC. All biochemical indexes of liver, kidney and fs-GLU were subjected to MRL analyses. All biochemical indexes data of them were statistically analyzed by using the SPSS software 17. The *p* < 0.05 was considered to indicate statistical significance in all analyses. In order to convince the correlation between fs-TG and fs-TC with liver, kidney and fs-GLU test. The variable importance in the projection (VIP) in modeling regression model was assessed by partial least squares analysis (PLS).

### BP-ANN prediction model

The fs-TG, fs-TC and relevant correlated biochemical indexes were employed into the BP-ANN. When developing fs-TG model, the correlated biochemical indexes were selected as the input layer, and the output layer was fs-TG level. When developing fs-TC model, the correlated biochemical indexes were selected as the input layer, and the output layer was fs-TC level. The transfer function of the hidden layers nodes and output layer nodes was tansig and purelin. The node numbers of hidden layer were calculated based on the formula of $m=\sqrt{n+ l}+ a$, where *m* is the number of the nodes in the hidden layer, and *n* is the number of nodes in the input layer, *l* is the number of nodes in the output layer, *a* is a constant from 1 to 10 [9, 16]. The BP-ANN model of fs-TG and fs-TC were established at Matlab R2011a.

## Results

### Characteristics of healthy and overweight

There are 302 healthy subjects and 273 overweight subjects enrolled in this study, the mean age were 40.34 ± 9.31 and 45.79 ± 11.06 year, BMI were 21.36 ± 1.44 and 26.01 ± 1.01. The Shapiro-Wilk test showed that most of indexes, except for TB and ALB, were abnormal distribution in two different groups. Thererfore, the difference of biochemical indexes between two groups was analyzed by Mann-Whitney method of Two-Independent-Sample-test in Nonparameter test, the results showed there was significant difference for all biochemical indexes (Table 1).

**Table 1:** Characteristics and difference of biochemical indexes in healthy and overweight

Index	Healthy		Overweight		*P*
	Mean	SD	Mean	SD	
fs-TG (mmol/L)	0.88	0.42	2.05	1.75	0.000
fs-GLU (mmol/L)	5.32	0.39	5.93	1.28	0.000
fs-LDL-c (mmol/L)	2.38	0.54	2.87	0.69	0.000
fs-HDL-c (mmol/L)	1.57	0.32	1.27	0.30	0.000
fs-TC (mmol/L)	4.40	0.63	5.00	0.91	0.000
fs-ALT (U/L)	15.31	6.99	33.39	22.60	0.000
fs-AST (U/L)	18.48	4.24	24.60	9.46	0.000
fs-GGT (U/L)	15.23	5.96	51.57	61.95	0.000
fs-TP (g/L)	75.74	3.79	76.58	3.58	0.009
fs-ALB (g/L)	46.69	2.47	47.42	2.68	0.000
fs-Cr (μmol/L)	55.08	10.14	69.30	14.47	0.000
fs-BUN (mmol/L)	4.68	1.07	5.29	1.20	0.000
fs-AKP (U/L)	64.62	17.34	80.68	21.78	0.000
fs-TBIL(μmol/L)	10.04	3.15	11.36	4.09	0.000
fs-DBIL(μmol/L)	3.28	1.11	3.60	1.41	0.012
fs-UA (μmol/L)	261.80	42.74	364.93	88.16	0.000

### Correlation analysis of TG, TC in healthy and overweight

Since these indexes were abnormal distribution, the correlation of TG and TC with indexes of weight, height, age, BMI, liver function, kidney function and fasting blood-glucose were analyzed by Spearman’s test. The correlation coefficient was used to identify the relationship between TG, TC and related indexes. The results showed there was different correlation of TG, TC in healthy and overweight. For example, fs-TC correlated with fs-GLU in healthy (*P* = 0.005), it lost correlation with fs-GLU in overweight (*P* = 0.064). In overweight, there was high relationship between fs-TG and weight, height, BMI, fs-GLU, fs-ALT, fs-AST, fs-GTT, fs-TB, fs-ALB, fs-CR, fs-AKP, fs-UA; while fs-TC was correlated with age, fs-ALT, fs-AST, fs-GTT, fs-DBIL (Table 2). The PLS was performed by using “eigs” to find a few eigenvalues and eigenvectors and “corrcoef” to calculate the correlation coefficients. The results showed there was similar correlation of TG, TC in healthy and overweight, the most important variables (VIP > 1) was fs-UA, followed by height, fs-TB, fs-AKP, fs-CR, weight, fs-ALB, age, which was consistent with the results of spearman’s test. The VIP figures of correlated indexes in modeling TG, TC regression model were showed in Additional file 1.

**Table 2:** Correlation coefficient of fs-TG and fs-TC with indexes of fs-GLU, liver and kidney in healthy and overweight

Index	Healthy				Overweight			
	fs-TG		fs-TC		fs-TG		fs-TC	
	Coefficient	*P*	Coefficient	P	Coefficient	*P*	Coefficient	*P*
Weight	.123*	0.033	0.011	0.854	.325**	0.000	-0.074	0.227
Height	-0.011	0.846	-0.099	0.085	.270**	0.000	-0.088	0.147
Age	.271**	0.000	.328**	0.000	0.006	0.922	.196**	0.001
BMI	.188**	0.001	.122*	0.033	.251**	0.000	0.025	0.681
fs-TG	1.000	-	.224**	0.000	1.000	-	.288**	0.000
fs-GLU	.123*	0.032	.162**	0.005	.157**	0.010	0.113	0.064
fs-TC	.224**	0.000	1.000	-	.288**	0.000	1.000	-
fs-ALT	.153**	0.008	.202**	0.000	.354**	0.000	.127*	0.037
fs-AST	0.072	0.215	.151**	0.008	.233**	0.000	.150*	0.013
fs-GTT	.248**	0.000	.151**	0.009	.505**	0.000	.241**	0.000
fs-TB	0.040	0.486	-0.020	0.729	.176**	0.004	.182**	0.003
fs-ALB	-0.047	0.415	0.017	0.773	.237**	0.000	0.050	0.415
fs-CR	.146*	0.011	0.018	0.762	.271**	0.000	-0.060	0.329
fs-BUN	-0.096	0.096	.188**	0.001	-0.063	0.299	0.034	0.574
fs-AKP	.220**	0.000	0.048	0.403	.221**	0.000	0.065	0.287
fs-TBIL	-0.039	0.498	0.002	0.968	0.117	0.054	-0.024	0.699
fs-DBIL	-.153**	0.008	-.273**	0.000	-0.080	0.192	-.281**	0.000
fs-UA	.163**	0.005	-0.026	0.653	.413**	0.000	0.077	0.209

^*^Correlation is significant at the 0.05 level (2-tailed)

^**^Correlation is significant at the 0.01 level (2-tailed)

### MRL analysis

According to the results of correlation analysis, fs-TG and fs-TC were related with indexes of liver and kidney. Considering the independent variables related to each other, MRL analysis was conducted by “stepwise” method to evaluate the biochemical indexes which were independently correlated to fs-TG and fs-TC.

The results showed six kinds of linear regression models generated, fs-ALT was the first variable involved in fs-TG linear regression models, and the other five indexes were fs-UA and fs-GTT. When fs-ALT, fs-UA, and fs-GTT involved in model, *R* = .407, Durbin-Watson test was 1.844, which indicated the distribution of residual was normal and the developed model was reliable. The contribution of each independent variable to fs-TG was showed in Table 3. The Beta values indicated fs-ALT, fs-UA, and fs-GTT have strongest contribution to fs-TG (*P* < 0.05), and these independent variables had no collinearity between each other.

**Table 3:** Regression coefficients of fs-TG, fs-TC MRL model based on independent variables

Model		Unstandardized coefficients		Standardized coefficients	t	Sig.	Collinearity statistics	
		B	Std. error	Beta			Tolerance	VIF
fs-TG	(Constant)	-.171	.421		-.405	.686		
	fs-ALT	.015	.005	.191	2.889	.004	.719	1.391
	fs-UA	.004	.001	.212	3.569	.000	.885	1.130
	fs-GTT	.004	.002	.133	2.019	.044	.718	1.392
fs-TC	(Constant)	2.151	1.119		1.922	.056		
	fs-DBIL	-.187	.036	-.290	-5.135	.000	.960	1.042
	fs-GTT	.003	.001	.234	4.198	.000	.981	1.019
	AGE	.015	.005	.182	3.246	.001	.970	1.030
	fs-TB	.035	.014	.136	2.463	.014	.995	1.005

As for fs-TC, there are four variable, fs-DBIL, fs-GTT, age, fs-TB, involved in fs-TC linear regression models, and *R* = .434, Durbin-Watson test was 2.011. The contribution of each independent variable to fs-TC was showed in Table 3.

### BP-ANN prediction model of fs-TG and fs-TC

**Fig. 1:**
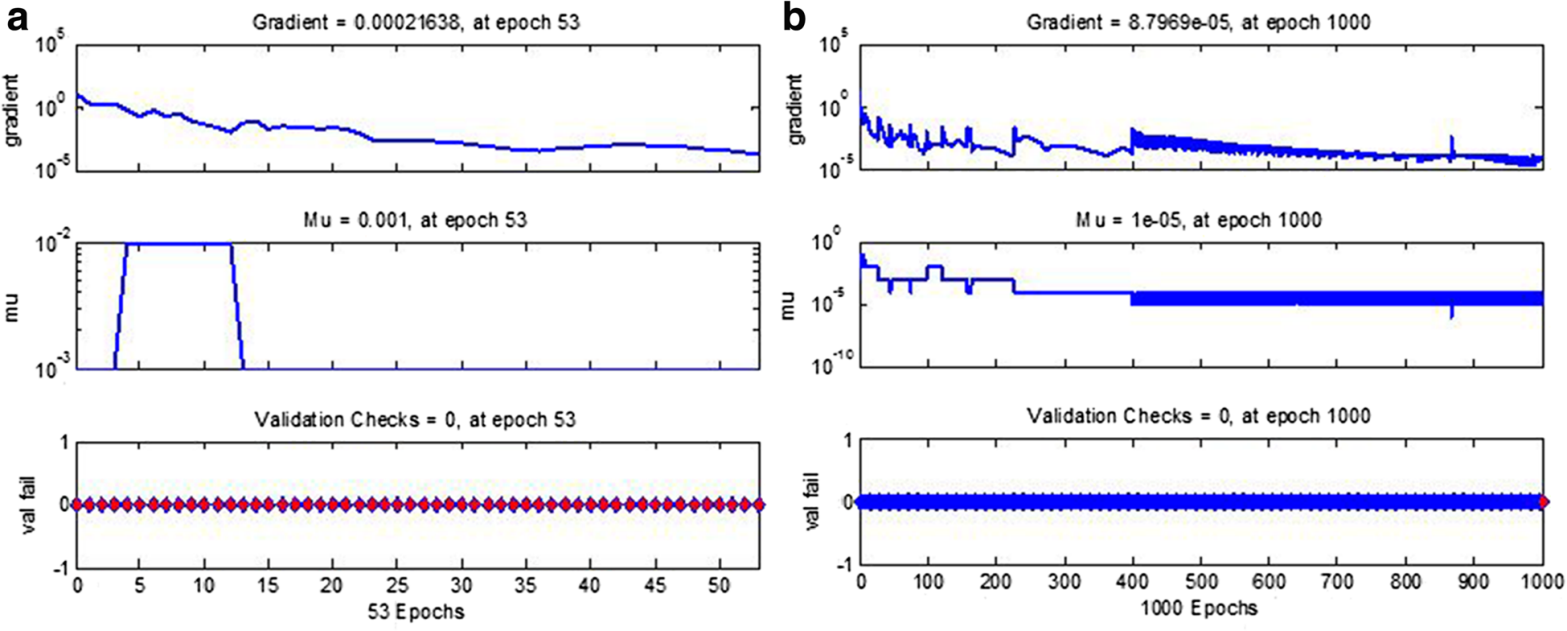
The fitness index of BP-ANN model of fs-TG achieved at epoch 53 (**a**), fs-TC achieved at epoch 1000 (**b**) performed in overweight people

**Fig. 2:**
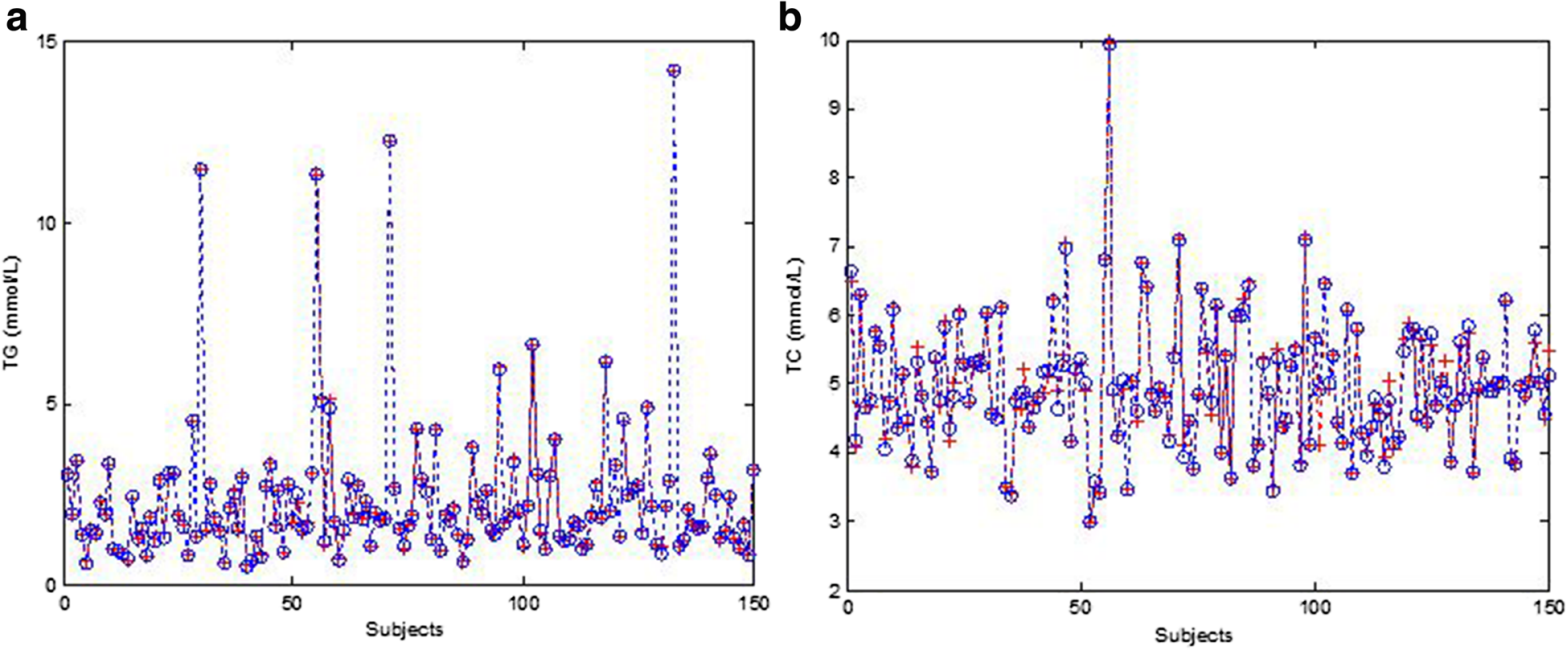
The measured concentrations (“+”) of fs-TG (**a**), fs-TC (**b**) and predicted concentrations (“o”) of fs-TG, fs-TC generated by BP-ANN Model in overweight people. The training goal was set at 1.5 × 10^-5^

According to the results of correlation analysis, weight, height, fs-ALT, fs-GTT, fs-CR, fs-AKP, fs-UA; fs-DBIL, fs-TBIL, and fs-ALB were selected as input layer, fs-TG was set as output layer. As for fs-TC, age, fs-ALT, fs-AST, fs-GTT, fs-TB, fs-DBIL, were selected as input layer. The training epoch was set at 1000, training goal was set at 0.5 × 10-5. After training, the BP-ANN model of fs-TG was reached the training goal at 53 epochs. The fs-TC model didn’t reach the goal, however, it showed high accuracy after training 1000 epochs. The performance of BP-ANN model of fs-TG and fs-TC were evaluated by mean square error (MSE), magnitude of the gradient, the number of validation checks, correlation coefficient. The best training performance of fs-TG was 4.7 × 10^-5^ at epoch 53 (*R* = 0.9997), the fs-TC was 1.1 × 10^-3^ at epoch 1000 (*R* = 0.9922). The gradient and validation checks of modeling fs-TG and fs-TC was showed in Fig. 1. The predicted and measured profiles of fs-TG and fs-TC in test group were showed in Fig. 2, which clearly indicated that the developed BP-ANN models were reached high accuracy in prediction of fs-TG and fs-TC.

## Discussion

Fasting TG and TC is very important index in clinical practice. The doctor can diagnose whether the patient had hyperlipemia according to the results of serum lipid and lipoprotein test. As overweight people have high risk for hyperlipidemia, which will cause heart attack or stroke [17], they should pay attention to TG and TC levels and monitor them frequently. However, the determination of TG and TC was separated from blood glucose, liver function, and kidney function test which are routinely monitored in clinical practice. The independent determination of TG and TC is inconvenient and increases the medical costs. Therefore, development of prediction model of TG and TC by indexes of blood glucose, liver function, and kidney function will be useful in clinical practice. And assessing the correlation of TG and TC with blood glucose, liver function, and kidney function test will be contribute to understand the mechanism of high dyslipidaemia, especially for overweight people.

In the present study, we developed prediction models of TG and TC according to the fasting (fs) biochemical indexes. Firstly, we analyzed the relationships between fs-TG, fs-TC and indexes of age, weight, height, BMI, blood glucose, liver function, and kidney function. The related parameters were included as independent variable for linear regression. The MRL showed only fs-ALT, fs-UA and fs-GGT were related to fs-TG, and fs-DBIL, fs-GTT, AGE, fs-TB were related to fs-TC, the other indexes were excluded from the regression model although they showed high correlation with fs-TG and fs-TC in correlation analysis.

Although the developed regression equation of MRL for fs-TG and fs-TC had statistical significance (*P* < 0.01), the multiple correlation coefficient of them were *R* = 0.407 and *R* = 0.434. In order to obtain more precise value of prediction, we introduced artificial neural network. Artificial neural network is a new modeling approach inspired by a brain’s central nervous system and has capable of machine learning, such as supervised learning, unsupervised learning and reinforcement learning. It has been used to diagnose diseases such as cancers or predict the outcome of treatment in medical area [18–20].

Back propagation is a kind of algorithms for training artificial neural networks. It is a fundamental and is a commonly used algorithm that instructs an ANN how to carry out a given task [21]. The predictive ability based on the correlation between input data and output data. In theory, the more relevant vector factors in input layer, the higher accuracy of prediction in BP-ANN Model [22].

In our study, when the training goal was set at 0.5 × 10^-5^, the BP-ANN model of fs-TG was far more quickly reached the goal than fs-TC and showed well accuracy. This is consistent with the correlation analysis of fs-TG and fs-TC, as fs-TG related to more biochemical indexes than fs-TC. Although, fs-TC didn’t reach the training goal within 1000 epochs, it still well performed (*R* = 0.9987). That indicated the BP-ANN model of fs-TC can also achieve high accuracy when selected age, fs-ALT, fs-AST, fs-GTT, fs-TB, fs-DBIL as input layer.

## Conclusion

There was high relationship of fs-TG and fs-TC with biochemical indexes, fs-TG was related to fs-GLU, fs-ALT, fs-AST, fs-GTT, fs-TB, fs-ALB, fs-CR, fs-AKP, fs-UA; while fs-TC was correlated with fs-ALT, fs-AST, fs-GTT, fs-DBIL (*P* < 0.01). Moreover, correlation analysis showed fs-TG was related to weight, height, BMI, while fs-TC was correlated with age. The MRL analysis indicated that only fs-ALT, fs-UA, and fs-GGT were related to fs-TG, while fs-DBIL, fs-GTT, AGE, and fs-TB were related to fs-TC. Based on correlation analysis, the BP-ANN models of fs-TG and fs-TC were developed which achieved high prediction accuracy and can be used to predict the level of fs-TG and fs-TC in blood.

## Additional file

Additional file 1: The Figure of VIP of correlated indexes in modeling TG, TC regression model generated by PLS algorithm. (DOCX 59 kb)

## Abbreviations

BP-ANN: Back propagation artificial neural network
fs-AKP: Alkaline phosphatase
fs-ALB: Albumin
fs-ALT: Alanine transaminase
fs-ALT: Aspartate aminotransferase
fs-BUN: Urea nitrogen
fs-Cr: Creatinine
fs-DBIL: Direct bilirubin
fs-GGT: Gamma-glutamyl transpeptidase
fs-GLU: Blood glucose
fs-TBIL: Total bilirubin
fs-TC: TC
fs-TG: Fasting indexes of TG
fs-TP: Total protein
fs-UA: Uric Acid
MRL: Multiple linear regression
TC: Cholesterol
TG: Triglyceride
WHO: World Health Organization
