# Supplementary material for: Assessment of triglyceride and cholesterol in overweight people based on multiple linear regression and artificial intelligence model
Source: Lipids Health Dis. 2017 Feb 20;16:42. doi: 10.1186/s12944-017-0434-5 (PMC5319080; doi:10.1186/s12944-017-0434-5)
Supplement: Additional file 1: — The Figure of VIP of correlated indexes in modeling TG, TC regression model generated by PLS algorithm. (DOCX 59 kb) [file 12944_2017_434_MOESM1_ESM.docx]

**Additional file 1**

The Figure of VIP of correlated indexes in modeling TG, TC regression model generated by PLS algorithm


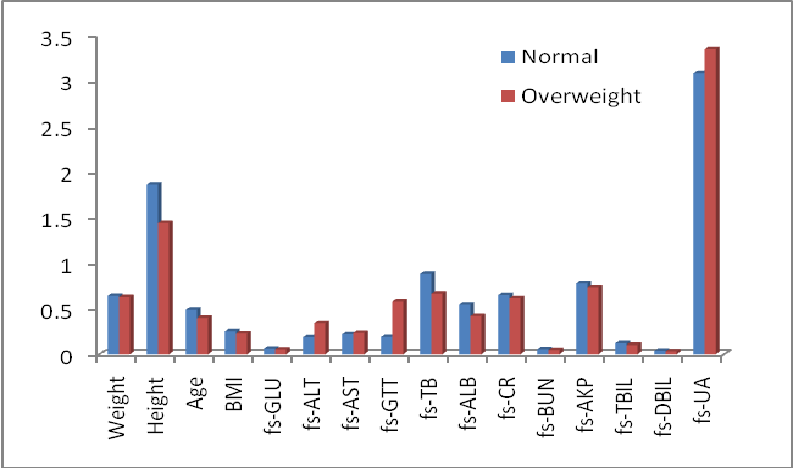


VIP of correlated indexes in modeling TG regression model generated by PLS algorithm


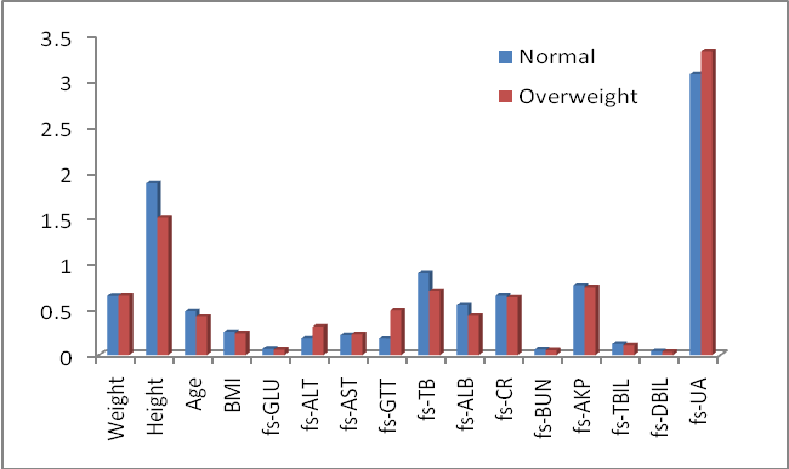


VIP of correlated indexes in modeling TG regression model generated by PLS algorithm
